# Diagnostic Value of Interferon-Gamma Release Assays Combined with Multiple Indicators for Tuberculous Peritonitis

**DOI:** 10.1155/2020/2056168

**Published:** 2020-03-20

**Authors:** Xidong He, Yuanxue Gao, Qi Liu, Zhifang Zhao, Wanhang Deng, Hong Yang

**Affiliations:** ^1^Department of Gastroenterology, The Affiliated Hospital of Guizhou Medical University, Guiyang 550004, China; ^2^Department of Gastroenterology, Guizhou Provincial People's Hospital, Guiyang 550002, China

## Abstract

**Objective:**

To investigate the diagnostic value of interferon-gamma release assays combined with multiple indicators for tuberculous peritonitis.

**Methods:**

Patients who were admitted to the hospital due to suspected tuberculous peritonitis were prospectively included during the 30-month study period. Moreover, healthy individuals were recruited and included in the control group. All the study participants were assessed using various indexes, such as interferon-gamma release assays.

**Results:**

A total of 180 patients with suspected tuberculous peritonitis were enrolled, and 24 were excluded. 73 patients with a confirmed diagnosis of tuberculous peritonitis were included in the tuberculous peritonitis group, 83 patients with other diseases in the other-disease control group, and 52 healthy individuals in the control group. Moreover, 83 patients in the other-disease control group and 52 participants in the control group were identified as 135 nontuberculous peritonitis patients. The area under the receiver operating characteristics curve for the QuantiFERON-TB test was 0.851 (95% confidence interval: 0.799–0.903), and the optimal cutoff value was 0.55 IU/mL, which corresponds to a sensitivity and specificity of 86.30% and 80.00%, respectively. The receiver operating characteristic curves for the combination of the QuantiFERON-TB test and the use of erythrocyte sedimentation rate, serum adenosine deaminase level, serum cancer antigen 125 level, and hypersensitive C-reactive protein level had an area under the curve of 0.859 (95% confidence interval: 0.809–0.909), with a sensitivity and specificity of 97.26% and 62.96%, respectively.

**Conclusions:**

The combined use of the QuantiFERON-TB test and multiple indexes can significantly improve the accuracy of diagnosing tuberculous peritonitis.

## 1. Introduction

In 2016, approximately 10.4 million new cases of tuberculosis (TB) were recorded worldwide. According to the World Health Organization (WHO), approximately 895,000 patients were newly diagnosed with TB in China in 2016, accounting for 8.6% of new cases globally [1]. The incidence rate of tuberculous peritonitis (TBP) is increasing, accounting for 6.1% of all extrapulmonary TB cases [2], and such condition is primarily observed in young individuals in developing countries in Asia and Africa [3]. Since tuberculin skin test is inexpensive and easy to perform, it has been widely used to diagnose TB, and its sensitivity and specificity are 66.5% and 63.3%, respectively. However, the population vaccinated with Bacillus Calmette-Guerin (BCG) and those with nontuberculous mycobacterium infection may present with false-positive test results, which lead to poor specificity [4, 5]. Thus, the diagnostic gold standard for TB is the culturing of *Mycobacterium tuberculosis* (MTB); however, the positive rate of ascites rapid smear for acid-fast bacilli is only 0%–6%. The positive rate of culturing MTB is higher. However, culturing will take 8 weeks [6, 7]. Thus, some patients must undergo peritoneal biopsy via laparoscopy to diagnose TBP [8], but for economically underdeveloped areas with a high incidence rate of TB, such procedure increases not only the health care costs of the patients but also the risk of developing different complications. Thus, the availability of peritoneal biopsy via laparoscopy is often limited. In addition, more studies should be performed to validate the efficacy of using ultrasound-guided peritoneal biopsy for the diagnosis of TBP [9]. Due to the atypical clinical manifestations of TBP and the low accuracy of diagnostic tests, its diagnosis becomes challenging, and this delays the onset of anti-TB treatment and leads to increased mortality. Therefore, a noninvasive, rapid, economical, and suitable TBP diagnostic method that can be used in areas with high incidence rates of TB must be urgently identified.

Interferon-gamma release assays (IGRAs) include the tuberculosis infection-specific cellular immune response (QuantiFERON-TB, QFT) and tuberculosis-infected T-cell spot test (T-SPOT.TB) [4]. IGRAs use the highly specific early secretory antigen target-6 (ESAT-6) and culture filtrate protein 10 as the antigens. The new generation of IGRAs also includes a third antigen (Rv2654 [TB7.7] or Rv3615c) [10]. For active TB, the sensitivity and specificity of T-SPOT.TB in the peripheral blood are 82.9% and 78.6%, respectively. The sensitivity and specificity of QFT are 81.7% and 75.2%, respectively. A previous study has shown that the use of IGRAs alone cannot be used for the diagnosis of active TB [11]. Cho et al. [12] have reported a sensitivity and specificity of 86% and 67% in the peripheral blood and 92% and 86% using the T-SPOT.TB for the diagnosis of TBP, respectively.

Adenosine deaminase (ADA), erythrocyte sedimentation rate (ESR), high-sensitivity C-reactive protein (hs-CRP), tuberculosis antibody (TB-Ab), and cancer antigen 125 (CA125) are commonly used for the diagnosis of TB [13–15].

The early diagnosis of TBP is challenging for clinicians. Researchers in Korea have proposed the combined use of T-SPOT.TB and ADA as an effective method for diagnosing TBP [16]. However, studies about the diagnostic value of the combined use of multiple indicators for TBP are limited. Therefore, the present study evaluated the diagnostic value of IGRAs combined with ESR, ADA, CA125, and hs-CRP for the diagnosis of TBP in areas that are at high risk for MTB infection.

## 2. Materials and Methods

### 2.1. Subjects

Adult and adolescent patients (≥14 years of age) who were admitted at the Affiliated Hospital of Guizhou Medical University between February 2015 and October 2017 due to suspected TBP were prospectively included in the study. The patients were suspected with a more typical TBP based on clinical signs and symptoms and/or imaging and ascites test results. In addition, healthy individuals were randomly selected and included in the control group (CON). The medical history of the enrolled participants was recorded, and physical examination was performed in detail. All patients completed the blood IGRA, ESR, ADA, CA125, hs-CRP, TB-Ab, and hepatitis virus detection, screening for AIDS and autoimmune diseases, as well as abdominal CT scan. Those with ascites were extracted to complete routine examination, ascites biochemistry, ascites ADA test, ascites CA125 test, and ascites culture, and some were willing to complete the peritoneal biopsy.

### 2.2. Grouping Criteria

1. TBP group: The definition of extrapulmonary TB in the 2010 WHO guidelines for TB treatment [17], the classification criteria for diagnosing TB by Liebeschuetz et al. [18], and the diagnosis standard for TBP by Cho et al. [12] were as follows: (1) confirmed cases: positive results for MTB culture using ascitic fluid or polymerase chain reaction (PCR), and (2) clinically diagnosed cases: typical clinical, imaging, and ascites characteristics of TBP and meeting either of the following: (1) peritoneal biopsy pathology result indicative of caseous granuloma or (2) undergoing anti-TB treatment (≥4–6 weeks), with signs and symptoms that significantly improved or were treated after 3 months of follow-up
2. Non-TBP group: patients in the other-disease control (ODC) and CON groups were combined into the non-TBP group
3. ODC group: patients with a confirmed diagnosis of other diseases and with either improved symptoms after (non-anti-TB) treatment or ineffective anti-TB treatment (after ≥4–6 weeks).
4. CON group: based on random sampling, individuals undergoing health examinations were included and classified into the CON group if no abnormality was found during examination

### 2.3. Exclusion Criteria

The following patients were excluded: (1) patients with inaccurate diagnosis; that is, patients who did not receive diagnostic anti-TB treatment were excluded when they still could not be accurately diagnosed at discharge, and those receiving diagnostic anti-TB treatment were excluded when their conditions did not improve after 4 weeks of anti-TB treatment; (2) those who were tested positive for human immunodeficiency virus (HIV) infection; (3) those with TB in other parts of the body who were undergoing anti-TB treatment within 2 weeks before admission; (4) those who were tested positive in bacteria (non-MTB) culture using ascitic fluid; (5) those with autoimmune disease; (6) those with uncertain IGRA results; and (7) those who were lost to follow-up or who died.

### 2.4. IGRAs

The QFT was tested using the QuantiFERON-TB Gold detection kit (ELISA, Cellestis, Victoria, Australia). T-SPOT.TB was tested using the TB infection T cell test kit (Immunization Spot Method, Oxford Immunotec, Abingdon, UK).

### 2.5. ESR and ADA, CA125, hs-CRP, and TB-Ab Levels

The ESR was tested using an automated rapid ESR analyzer Roller 20 (Alifax, Padova, Italy). ADA levels were tested using an adenosine deaminase detection kit (peroxidase method, Jiuqiang Biotech Co., Ltd., Beijing, China). CA125 levels were tested using a quantitative assay kit (electrochemiluminescence method, Roche Diagnostics GmbH, Mannheim, Germany). hs-CRP levels were tested using a protein assay kit (particle-enhanced immunity transverse turbidity method, DiaSys Diagnostic Systems GmbH, Holzheim, Germany). TB-Ab levels were tested using an antibody kit (Bell Bioengineering Co., Ltd., Beijing, China).

### 2.6. Statistical Analysis

Statistical analysis was performed using the IBM Statistical Package for the Social Sciences software for Windows version 20.0: comparison among the TBP, ODC, and CON groups, meanwhile comparing the TBP with non-TBP groups. Quantitative data with normal distributions were expressed as means ± standard deviation($\bar{x}\pm s$). Comparisons among the three groups were performed via analysis of variance. Moreover, comparisons between the two groups were carried out using the least significant difference (LSD) method. The quantitative data of skewed distributions were expressed as P50 (P25, P75), and their differences were tested using the Mann–Whitney *U* test, nonparametric Kruskal–Wallis *H* test for the three groups, and Nemenyi test for pairwise comparisons. Qualitative data were expressed as rates (%). Chi-square tests were used for comparisons among the three groups, and the chi-square segmentation method was used for comparisons between the two groups. A *p* value < 0.05 was considered statistically significant. The receiver operating characteristic (ROC) curves were used to assess the diagnostic value of the QFT alone or in combination with ESR, ADA, CA125, and hs-CRP for the diagnosis of TBP because the T-SPOT.TB was a qualitative datum and no ROC curve was obtained. The indicators involved in the combined detection were analyzed using logistic regression, and the combined prediction probability was calculated. Then, the ROC curve of the combined detection was obtained with this probability. The area under the curve (AUC) was then calculated. The cutoff point corresponding to the maximum Yoden index was set as the best diagnostic clinical cutoff value to calculate the sensitivity and specificity. Z tests were used to compare the AUCs of different indicators, and a *p* value < 0.05 was considered statistically significant.

## 3. Results

### 3.1. Inclusion and Grouping

**Figure 1:**
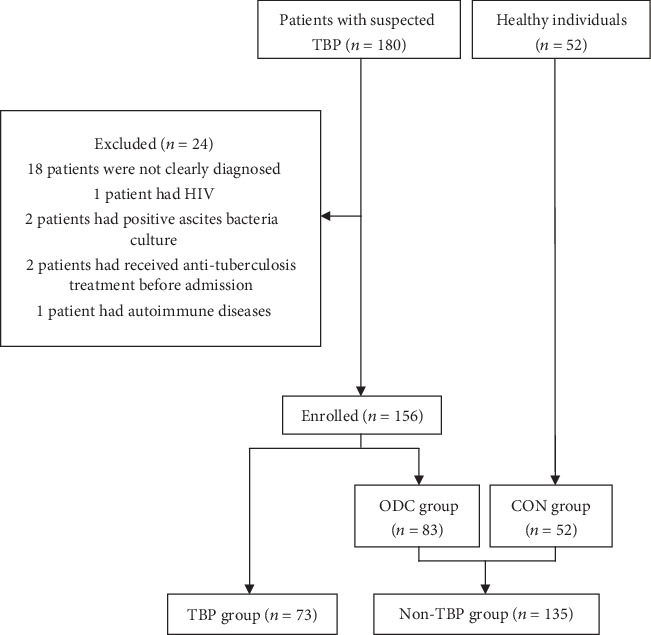
Flowchart of patient selection. n: number of patients; TBP: tuberculous peritonitis; ODC: other-disease control; CON: control.

**Figure 2:**
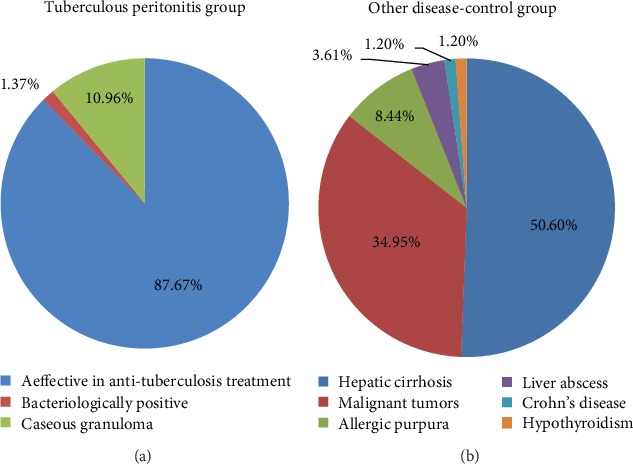
Diagnostic compositions of three groups TBP and group ODC.

A total of 180 patients with suspected TBP were enrolled. Of these patients, 24 were excluded after diagnosis (18 patients were not accurately diagnosed, one patient had HIV, two patients had positive ascites bacteria culture, two patients had received anti-TB treatment before admission, and one patient had autoimmune diseases). A total of 73 patients were finally included in the TBP group, 83 patients in the ODC group, and 52 in the CON group (Figure 1). One patient in the TBP group had positive bacteriology results. A total of 11 patients underwent biopsy, and eight had pathological findings suggestive of caseous granuloma. Of the 8 patients, six and two patients underwent laparoscopic peritoneal biopsy and ultrasound-guided peritoneal biopsy, respectively. A total of 64 patients were diagnosed with TBP after an effective anti-TB treatment. In the ODC group, 7 patients presented with abdominal allergic purpura, 42 with hepatic cirrhosis, 29 with various malignant tumors, 3 with liver abscess, 1 with Crohn's disease, and 1 with hypothyroidism (Figure 2).

### 3.2. Comparison of T-SPOT.TB, QFT, ESR, ADA (Serum and Ascites), CA125 (Serum and Ascites), hs-CRP, TB-Ab, and Clinical Characteristic between the TBP and Non-TBP Groups

In this study, the non-TBP group, composed of the ODC and CON groups, included 135 patients. The participants in the TBP group were younger than those in the non-TBP group (*p* < 0.05). No significant difference was observed in terms of sex between the TBP and non-TBP groups (*p* > 0.05). The median QFT, ESR, serum ADA level, serum CA125 level, and hs-CRP values in the TBP group were significantly higher than those in the non-TBP group (*p* < 0.05), and the T-SPOT.TB and TB-Ab positive rates were higher in the TBP group than in the non-TBP group (*p* < 0.05). The participants in the TBP group were younger than those in the ODC group and CON group (*p* < 0.05). The median QFT and serum ADA, hs-CRP, and ascitic ADA levels and positive rates of the T-SPOT.TB and TB-Ab were higher in the TBP group than those in the ODC group (*p* < 0.05). However, the median ascitic CA125 level was lower in the TBP group than in the ODC group (*p* < 0.05). The median ESR and serum CA125 level did not significantly differ between the TBP and ODC groups (*p* > 0.05). The median QFT, ESR, serum ADA level, serum CA125 level, and hs-CRP level as well as T-SPOT.TB and TB-Ab positive rates in the TBP group were higher than those in the CON group (*p* < 0.05). The median ESR, serum ADA level, serum CA125 level, and hs-CRP levels as well as T-SPOT.TB positivity rates in the ODC group were higher than those in the CON group (*p* < 0.05); however, the median QFT did not significantly differ between the ODC and CON groups (*p* > 0.05), and the TB-Ab positivity rate in the ODC group was lower than that in the CON group (*p* < 0.05) (Table 1).

### 3.3. ROC Curves and Optimal Cutoff Values of QFT, ESR, ADA (Serum and Ascites) Level, CA125 (Serum and Ascites) Level, and Hs-CRP Level

**Figure 3:**
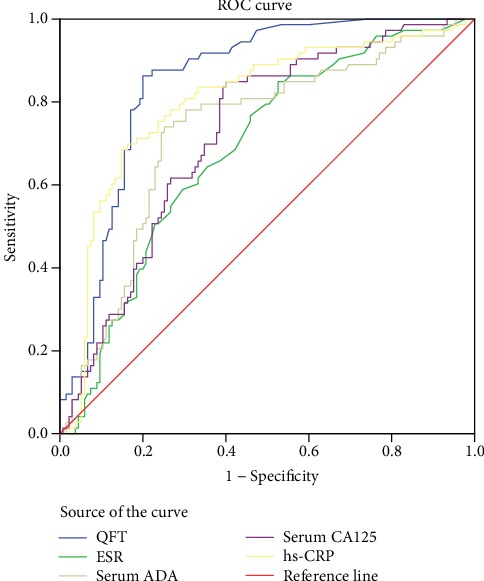
ROC curves of different various quantitative tests. QFT: Quanti FERON-TB; ESR: erythrocyte sedimentation rate; ADA: adenosine deaminase; CA125: cancer antigen 125; hs-CRP: high-sensitivity C-reactive protein.

**Figure 4:**
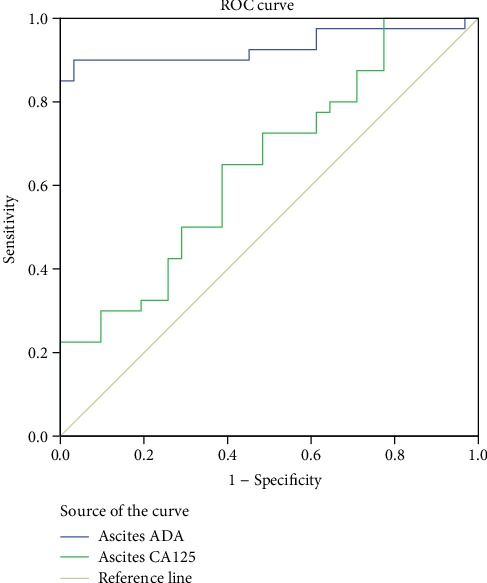
ROC curves of ascitic ADA and ascetic CA125. ADA: adenosine deaminase; CA125: cancer antigen 125.

The AUC of the QFT curve was 0.851 (95% confidence interval [CI]: 0.799–0.903), which was higher than that of ESR and serum ADA, serum CA125, and hs-CRP levels (*p* < 0.05). The optimal cutoff value of the QFT was 0.55 (IU/mL), with a sensitivity and specificity of 86.30% and 80.00%, respectively (Figure 3, Table 2). The ROC AUC of ascitic ADA level was 0.932 (95% CI: 0.866–0.998), which was higher than that of ascitic CA125 level (*p* < 0.05) (Figure 4, Table 2).

### 3.4. ROC Curves and Diagnostic Values of QFT Combined with ESR and Serum ADA, Serum CA125, and Hs-CRP Levels (Two, Three, Four, or Five Items in Combination)

**Figure 5:**
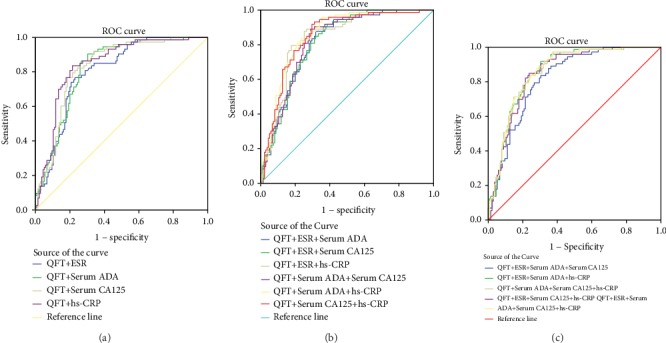
ROC curves of various combination methods combined detection. QFT: Quanti FERON-TB; ESR: erythrocyte sedimentation rate; ADA: adenosine deaminase; CA125: cancer antigen 125; hs-CRP: high-sensitivity C-reactive protein. (a) two-tem in combination; (b) three-tem in combination; (c) four- and five-tem in combination.

The AUCs of the ROCs for various combined methods were >0.8. The AUC of the ROCs for the combined five items was 0.859 (95% CI: 0.809–0.909), with a sensitivity and specificity of 97.26% and 62.96%, respectively (Figure 5, Table 3).

### 3.5. Diagnostic Performance of T-SPOT.TB, QFT (≥0.55 IU/mL), and Ascitic ADA Level

The positive and negative predictive values were further calculated for T-SPOT.TB, QFT (≥0.55 IU/mL), and ascitic ADA level, which had higher diagnostic accuracies (Table 4).

## 4. Discussion

About one-third of the world's population is infected with MTB [19]. TBP is primarily observed in young adults aged between 30 and 50 years [20–22]. The primary symptom is abdominal pain [23, 24].

This study diagnosed 73 patients with TBP, of which one (1.37%) presented with bacteriologically positive TB. Because the number of bacteria in peritoneal effusion or peritoneal tissue is extremely low, the sensitivities of acid-fast staining, MTB culture, and nucleic acid detection are significantly low to meet clinical needs [7, 25, 26]. In some cases, the diagnosis of TBP may require invasive surgery, such as laparoscopic peritoneal biopsy. Among the 73 patients with TBP in this study, only the diagnosis of eight (10.96%) patients was confirmed via pathological examination of the caseous granuloma because of the economic and surgical complications and other reasons. Although the positive rate of peritoneal biopsy is considerably high [3], the rate in this study was only 72.73% (8/11). However, its use is generally limited due to surgical complications, contraindications, or medical costs [8].

In developing countries and those with high incidence rates of TB, the diagnostic value of IGRAs differs from that in developed countries due to the large base of latent TB infection; in addition, the diagnostic value of IGRAs may also differ for TB in different parts of the body. Meanwhile, the value of IGRAs combined with other indicators for the diagnosis of tuberculous peritonitis also needs to be evaluated.

This study investigated the diagnostic value of IGRAs combined with multiple indexes often used in clinical settings for the diagnosis of TBP in high-risk areas. In our study, the patients in the TBP group were younger than those in the non-TBP group (39.05 ± 19.44 vs. 52.57 ± 15.49 years, *p* < 0.05), which is consistent with previous reports showing that TBP is more common in young and middle-aged patients [27, 28]. In the present study, although the ascitic CA125 level was also elevated in the TBP group, the median was lower than that of the ODC group (*p* < 0.05); this difference may be attributed to the presence of malignant diseases that increase the ascitic CA125 level; therefore, in the clinical setting, patients with malignant tumors should be excluded when the ascitic CA125 level is used in the diagnosis of TBP [29]. No significant difference was observed in ESR and serum CA125 levels between the TBP and ODC groups (*p* > 0.05). However, an increase may indicate the activity of TB and can facilitate in the diagnosis of TBP. The sensitivity of CA125 in this study was 84.93%, which was consistent with that of previous studies. The sensitivity of ESR was 84.93%, which is higher than that of previous studies at 74.74%. The positive rate of T-SPOT.TB was 94.52%, which is higher than that of previous studies at 82%. The TB-Ab rate in the previous study was 14.29%, whereas that in our study was 6.85% [30].

The ROC curve analysis revealed that the AUC of the ROC curve of the QFT was larger than those of ESR, serum ADA level, serum CA125 level, and hs-CRP level (*p* < 0.05). QFT can be used as the diagnostic index for TB peritonitis. The optimal cutoff value of QFT calculated using the Yoden index was 0.55 IU/mL, which is higher than the current global standard (0.35 IU/mL); this difference is more likely attributed to the high incidence and large base of latent TB infection in the area of this study [31, 32]. When the cutoff of QFT in cavitary pulmonary TB is 0.818 IU/mL, it can maximize the specificity without significant loss of test sensitivity [33]. In a study about the TB eye disease, the cutoff of QFT was 2 IU/mL, indicating that the cutoff value that was higher than that provided by the manufacturer should be considered to better identify ocular inflammation that is beneficial for full anti-TB treatment [34]. For the diagnosis of TB pleurisy, QFT had the best performance with a cutoff point of 2.33 IU/mL [35]. Another study about the diagnosis of tuberculous pleurisy pointed out that the optimal cutoff of QFT was 0.73 IU/mL for TB Gold assay in blood assay, 0.82 IU/mL for the cultured pleural fluid assay, and 0.94 IU/mL for isolated pleural cell assay [36]. No studies have investigated about the cutoff value of QFT for TBP. Thus, more studies about the optimal cutoff value of QFT in different conditions must be conducted [37]. The clinical cutoff value of QFT provided in the manual (0.35 IU/mL) is primarily used for the diagnosis of latent TB infection in countries with a low incidence of TB. The optimal QFT cutoff value in the present study (0.55 IU/mL) may be more conducive to the diagnosis of TBP in regions with high incidence rates of TB. Meanwhile, future studies about the optimal cutoff value of QFT must be conducted to distinguish latent and active TB infections in areas with high incidence rates of TB.

In terms of combined detection, regardless of the number of items in combination with QFT (two, three, four, or five items), the AUCs of the ROC curves were >0.8, indicating that various combined methods had high accuracy for the diagnosis of TBP. The negative predictive value of 3 or more combined detections ranged from 88.7% to 97.7%. In clinical practice, the diagnosis of tuberculous peritonitis can be basically ruled out, as 3 or more indices, including QFT, are negative. When QFT was combined with ESR, serum ADA level, serum CA125 level, and hs-CRP level, the AUC of the ROC curves was 0.859 (95% CI: 0.809–0.909), with a sensitivity as high as 97.26%, indicating the high diagnostic value for diagnosing TBP; however, the specificity was only 62.96%, which may be due to the large base of latent TB infection in this region [31, 32] and the low specificities of other combined traditional indicators. QFT + serum CA125 level has a specificity of 81.48%, a sensitivity of 76.71%, a positive predictive value of 69.1%, and a negative predictive value of 86.6%. Since it is impossible to calculate the cutoff value of each index or the total cutoff value of all indices using the combined detection, and the cutoff value of each index obtained from the individual test cannot be used for the combined detection, the cutoff value of each index in the specification should be taken as reference when using the combined detection in clinical work. When both QFT and CA125 are positive or significantly elevated, combining the medical history, signs and symptoms, and imaging, and/or ascites results, then the diagnosis of TBP and antituberculous therapy may be considered. The results of this study showed that the QFT combined with several other indicators as well as medical history and clinical manifestations is useful for the diagnosis of TBP. In clinical settings, we may consider the diagnosis of TBP when all five indices are positive, combining the patient's signs and symptoms as well as imaging and/or ascites results. If all the five indices are negative based on a negative predictive value of 97.7%, TBP may be ruled out. Moreover, the medical costs of these indicators are lower than those of surgery and without the occurrence of complications.

The AUC of the ROC curve for ascitic ADA level was 0.932 (95% CI: 0.866–0.998), indicating that ascitic ADA is a good indicator for the diagnosis of exudative TBP. Lee et al. [16] pointed out that the combined mode of T-SPOT.TB and ADA can guide the clinical diagnosis of tuberculous peritonitis. In the future, the study on the diagnosis of tuberculous peritonitis by ascites ADA should be strengthened, and more combined diagnosis modes should be provided.

This study was conducted in economically underdeveloped areas with a high incidence of tuberculosis; in this condition, there are many limitations in clinical peritoneal biopsy, and the positive rate of mycobacterium tuberculosis culture is low. Therefore, most patients in the TBP group only had clinical diagnosis, without diagnosis of histology and bacteriology. However, this is also consistent with the study objective of our clinical work on how to quickly diagnose tuberculous peritonitis. When using the combined test, there is no a total cutoff value, and the cutoff value of each indicator also cannot be estimated. The cutoff value of each index obtained from the individual test cannot be used for combined test, so this study only evaluated the diagnostic value of various combined test modes and the cutoff value of each index when using individual test, while we could not calculate the score or cutoff value of the combined test. Therefore, future study should design more diagnostic modes in order to guide clinical practice in a better way. This study calculated the optimal cutoff value of the QFT to diagnose TBP in areas with high incidence rates of TB. In addition, the innovative method of combining IGRAs with multiple indicators is helpful for the clinical diagnosis of TBP. Future studies must be conducted to assess the diagnostic value of IGRAs in ascites for TBP and provide a combination of multiple indicators for the diagnosis of TBP.

## 5. Conclusions

In areas with a high incidence of TB, QFT with a cutoff value of 0.55 IU/mL has a better diagnostic value for TBP. Furthermore, the combination of QFT as well as ESR, serum ADA level, serum CA125 level, and hs-CRP level can significantly improve the diagnostic accuracy for TBP. In particular, an increase in negative predictive values can be used to screen for TBP.

## Figures and Tables

**Table 1:** Comparison of baseline clinical characteristics and the results of various tests among various groups.

Clinical characteristic	TBP (*n* = 73)	Non-TBP (*n* = 135)		
		ODC (*n* = 83)	CON (*n* = 52)	Total (*n* = 135)
Age (years)	39.05 ± 19.44	54.20 ± 17.80^∗^	49.96 ± 10.51^∗^	52.57 ± 15.49^&^
Male (%)	34 (46.58)	52 (62.65)^∗^	15 (28.85)^∗^^#^	67 (49.62)
Median of ESR mm/H (IQR)	36.00 (21.50, 55.00)	30.00 (18.00, 49.00)	7.00 (3.25, 12.00)^∗^^#^	19.00 (7.00, 34.00)^&^
Median of serum ADA U/L (IQR)	20.47 (14.04, 28.81)	12.91 (8.84, 25.30)^∗^	9.50 (7.26, 10.39)^∗^^#^	10.36 (8.56, 15.70)^&^
Median of hs-CRP mg/L (IQR)	87.48 (27.25, 106.90)	19.61 (4.49, 43.28)^∗^	1.03 (0.32, 4.95)^∗^^#^	6.33 (1.04, 25.11)^&^
Positive TB-Ab (%)	5 (6.85)	0 (0.00)^∗^	1 (1.92)^∗^^#^	1 (0.74)^&^
Positive T-SPOT.TB (%)	69 (94.52)	32 (38.55)^∗^	8 (15.38)^∗^^#^	40 (29.63)^&^
Median of QFT IU/mL (IQR)	1.9 (0.76, 3.43)	0.05 (-0.01, 0.64)^∗^	0.02 (-0.01, 0.16)^∗^	0.02 (-0.01, 0.24)^&^
Median of serum CA125 U/mL (IQR)	223.40 (57.40, 449.72)	135.89 (18.61, 399.34)	8.59 (6.09, 12.05)^∗^^#^	18.81 (8.61, 183.03)^&^
Ascites extract (%)	40 (54.79)	31 (37.35)	0 (0.00)	—
Median of ascitic CA125 U/mL (IQR)	691.30 (398.72, 1212.58)	1071.00 (564.89, 1879.34)^∗^	—	—
Median of ascitic ADA U/L (IQR)	55.89 (48.68, 69.96)	5.81 (2.25, 10.07)^∗^	—	—

Note: ^∗^Compared with group TBP, *p* < 0.05, ^#^compared with group ODC, *p* < 0.05, ^&^compared with group TBP, *p* < 0.05. *n*: number of patients; TBP: tuberculous peritonitis; ODC: other-disease control; CON: control; ESR: erythrocyte sedimentation rate; ADA: adenosine deaminase; hs-CRP: high-sensitivity C-reactive protein; TB-Ab: tuberculosis antibody; T-SPOT.TB: tuberculosis-infected T-cells spot test; QFT: Quanti FERON-TB; CA125: cancer antigen 125.

**Table 2:** Comparison of baseline characteristics and optimal cutoff values of ROC curves between various quantitative tests.

Test	AUC	*p*	AUC 95% CI		Cutoff value	Sensitivity (%)	Specificity (%)
			Lower limit	Upper limit			
QFT	0.851	<0.001	0.799	0.903	0.55	86.30	80.00
ESR	0.686	<0.001	0.612	0.759	16.50	84.93	47.41
Serum ADA	0.726	<0.001	0.653	0.800	15.31	73.97	74.81
Serum CA125	0.723	<0.001	0.653	0.793	30.34	84.93	60.00
hs-CRP	0.802	<0.001	0.736	0.867	43.54	68.49	85.19
Ascitic ADA	0.932	0.034	0.866	0.998	24.06	90.00	96.77
Ascitic CA125	0.649	0.032	0.521	0.777	873.15	65.00	61.29

Note: Comparison of AUCs of ESR, serum ADA, serum CA125, and hs-CRP with QFT AUC, *p* < 0.05. Comparison of AUCs of ascitic ADA with ascitic CA125, *p* < 0.05. AUC: area under the curve; QFT: Quanti FERON-TB; ESR: erythrocyte sedimentation rate; ADA: adenosine deaminase; CA125: cancer antigen 125; hs-CRP: high-sensitivity C-reactive protein.

**Table 3:** Comparison of baseline characteristics and diagnostic value of ROC curves of various combined detection.

Combined detection	AUC	Sensitivity (%)	Specificity (%)	95% CI	PPV (%)	NPV (%)
Two-item in combination						
QFT + ESR	0.804	76.71	77.04	0.745-0.863	64.4	86.0
QFT + serum ADA	0.823	90.41	69.63	0.767-0.879	61.7	93.1
QFT + serum CA125	0.830	76.71	81.48	0.774-0.886	69.1	86.6
QFT + hs − CRP	0.844	83.56	78.52	0.789-0.898	67.8	89.8
Three-item in combination						
QFT + ESR + serum ADA	0.816	87.67	68.15	0.759-0.873	59.8	91.1
QFT + ESR + serum CA125	0.814	90.41	63.70	0.758-0.871	57.4	92.5
QFT + ESR + hs − CRP	0.840	87.67	74.81	0.785-0.894	65.3	91.8
QFT + serum ADA + serum CA125	0.823	89.04	71.11	0.767-0.879	62.5	92.3
QFT + serum ADA + hs − CRP	0.858	95.89	64.44	0.808-0.908	59.3	96.7
QFT + serum CA125 + hs − CRP	0.851	91.78	70.37	0.798-0.904	62.6	94.1
Four-item in combination						
QFT + ESR + serum ADA + serum CA125	0.819	83.56	69.63	0.763-0.875	59.8	88.7
QFT + ESR + serum ADA + hs − CRP	0.857	91.78	69.63	0.807-0.908	62.0	94.0
QFT + serum ADA + serum CA125 + hs − CRP	0.858	83.56	76.30	0.807-0.908	65.6	89.6
QFT + ESR + serum CA125 + hs − CRP	0.849	84.93	76.30	0.796-0.901	66.0	90.4
Five-item in combination						
QFT + ESR + serum ADA + serum CA125 + hs − CRP	0.859	97.26	62.96	0.809-0.909	58.7	97.7

Note: Comparison of AUCs of two-item in combination, *p* > 0.05; comparison of AUCs of three-item in combination, *p* > 0.05; comparison of AUCs of four-item in combination, *p* > 0.05. PPV: positive predictive value; NPV: negative predictive value; AUC: area under the curve; QFT: Quanti FERON-TB; ESR: erythrocyte sedimentation rate; ADA: adenosine deaminase; CA125: cancer antigen 125; hs-CRP: high-sensitivity C-reactive protein.

**Table 4:** Diagnostic performance of T-SPOT.TB, QFT (≥0.55 IU/mL), and ascitic ADA.

Assay item	Sensitivity (%)	Specificity (%)	Positive predictive value (%)	Negative predictive value (%)
T-SPOT.TB	94.52	70.37	63.3	96.0
QFT (≥0.55 IU/mL)	86.30	80.00	70.0	91.5
Ascitic ADA	90.00	96.77	97.3	88.2

Note: T-SPOT.TB: tuberculosis-infected T-cells spot test; QFT: Quanti FERON-TB; ADA: adenosine deaminase.

## Data Availability

The research data used to support the findings of this study are available from the corresponding author upon request.
